# Advances in Living Myocardial Slice Technology for Heart Failure Research

**DOI:** 10.1007/s11897-026-00762-z

**Published:** 2026-05-13

**Authors:** Azra Husetić, Anke M. Smits, Monika M. Gladka

**Affiliations:** 1 Department of Medical Biology, Amsterdam Cardiovascular Sciences, Amsterdam University Medical Centers, University of Amsterdam, Amsterdam, The Netherlands; 2 Department of Cell and Chemical Biology, Leiden University Medical Center, Leiden, The Netherlands

**Keywords:** Living myocardial slices, Heart failure, Disease modeling, Drug screening, Ex vivo model

## Abstract

**Purpose:**

The development of effective treatments for heart failure (HF) often fails due to the lack of preclinical models that closely reflect the native structure and function of the human myocardium. Living myocardial slices (LMS) are ultra-thin sections of heart tissue that have shown to retain the complexity, multicellularity, and function of the innate adult myocardium. The number of studies using LMS for HF research and its underlying diseases has been increasing rapidly over the last few years, mainly due to methodological advances that have prolonged LMS culture. This review summarizes key findings and various applications of LMS in HF research.

**Recent Findings:**

LMS derived from both animal and human hearts, including end-stage HF explants, donor hearts, or surgical specimens, have increasingly been used to model HF and related cardiac diseases. Moreover, LMS have enabled the study of human-specific responses to potential therapeutic drugs and replicate other drug-related effects, such as cardiotoxicity, as they appear in the clinic. Additionally, they have been used to validate the impact of gene delivery of pro-regenerative targets previously investigated in animal studies. More recently, LMS platforms have also been used to mimic device therapy for HF patients by controlling the mechanical and electrical parameters of LMS in culture.

**Summary:**

LMS represent a physiologically relevant model that bridges the gap between conventional in vitro systems and in vivo models in HF research. Despite remaining challenges related to tissue availability and culturing, LMS provide a highly translational platform for testing potential treatment strategies and understanding the underlying mechanisms of HF.

**Graphical Abstract:**

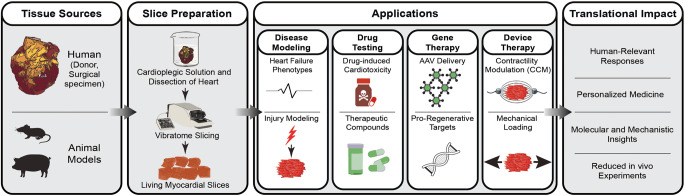

## Introduction

Heart failure (HF) remains one of the leading causes of morbidity and mortality globally, affecting more than 64 million people [1]. HF is a complex clinical syndrome that can arise from a wide spectrum of cardiac diseases, leading to structural remodeling and loss of functional properties. Despite considerable advances in translational and clinical research, developing effective therapies remains challenging in part due to the lack of preclinical models that accurately recapitulate the architecture, multicellularity, and disease phenotypes of the adult human heart. Even though significant progress has been made in developing in vitro models such as engineered heart tissue, cardiac organoids and other iPSC-derived models, these platforms still fail to recapitulate the complex, mature environment of the adult human myocardium. Therefore, there is a need for more physiologically relevant 3D models that can be applied in translational HF research.

Living myocardial slices (LMS), also referred to as cardiac or organotypic heart slices, are ultra-thin sections of heart tissue (approximately 100–400 μm) that overcome many limitations of conventional in vitro models. Multiple studies have demonstrated that LMS preserve the native three-dimensional tissue architecture of the living myocardium by maintaining intricate cell–cell interactions and extracellular matrix composition [2, 3]. Furthermore, LMS maintain the native cardiac function, enabling investigations into dynamic processes such as electrical activity and contractility [3, 4]. In this way, LMS closely mimic the complex in vivo conditions of the heart, providing a powerful platform for investigating cardiac physiology, pathophysiology, and pharmacological responses. Importantly, LMS can be prepared from several species, including human heart tissue. It has been shown that LMS prepared from HF patient-derived failing hearts retain the patient-specific characteristics, making them a personalized ex vivo model [5]. In recent years, the number of studies using LMS for various applications, including disease modeling, drug testing and pro-regenerative therapies, has been rising consistently and rapidly.

In this review, we summarize the historical development and methodological principles of LMS, with emphasis on recent advances in culturing methods. We then discuss the use of LMS to model HF and underlying cardiac diseases, followed by their application in pharmacological testing, including anthracyclines-induced cardiotoxicity, and therapeutic drugs. Finally, we provide an overview of how LMS have advanced HF research over the recent years.

### History of Living Myocardial Slices

The earliest report on cardiac slices dates back to a study in 1933, where excised rat tissues, including cardiac ventricular specimens, were used to investigate changes in oxygen consumption across different tissues after drug treatment [6]. At that time, cardiac slices were prepared manually using hand-held blades or razors. The quality of tissue sections improved significantly when high-precision vibratomes were introduced around the 1970s, which enabled precision-cut slices and reduced mechanical damage during the cutting procedure by providing control over slice thickness, blade movement speed, and amplitude [7]. Despite these technical advances, early studies were limited by the short-time viability of myocardial slices in culture [8]. This is because they were mostly cultured by submersion in culture medium, which was sufficient for maintaining LMS in culture for hours to a few days, before dedifferentiation of the tissue and loss of viability and structural integrity [9, 10]. A major breakthrough in prolonged LMS culture was achieved in 2012 with the introduction of a new culturing method by Brandenburger and colleagues. In this approach, slices are placed on a semipermeable transwell membrane above the culture medium, thereby creating an air-liquid interface, allowing efficient oxygenation of the tissue, and ultimately extending the culturing period to up to a week [11]. Air-liquid interface culturing is widely used and remains suitable for studies that do not require LMS culture for more than a week and that are based on molecular analysis without functional readouts [12, 13]. Since air-liquid interface culturing does not include mechanical loading or electrical stimulation of the slices, it leads to a faster reduction in contractile force, which hampers studies that aim to monitor slices for an extended time period. To address this limitation, Fischer and colleagues introduced the first commercially available biomimetic culturing system in 2019 [14]. This system incorporates auxotonic mechanical loading and continuous electrical pacing, resulting in sustained contractions of LMS and prolonged culture for several weeks. Since then, their tissue culturing system, “MyoDish” (InVitroSys GmbH, Germany), has been used by many groups for a range of applications that involve monitoring and stimulating contractility and electrophysiology in LMS [5, 14–27]. A comprehensive protocol for long-term LMS culture using the MyoDish system was published by Hamers et al. in 2022 [19]. At present, this biomimetic chamber system represents the most reliable approach for preserving functional viability of LMS by continuously monitoring their contractile activity. However, despite this highly significant advancement in LMS culture, the slices are still undergoing remodeling to some extent, indicating a need for optimized culturing systems for long-term studies, which remains a central topic in current discussions on the future development of LMS models [3].

### Preparation of LMS

The preparation of LMS is a critical determinant of tissue viability and functionality. LMS are most commonly generated from the ventricular myocardium, but other studies have shown that slices can also be generated from the epicardium and atria [28, 29]. Moreover, LMS have been successfully produced from a wide range of species, including mice [13, 30–35], rats [36–42], guinea pigs [43–45], rabbits [43, 46], dogs [13, 35, 47, 48] and pigs [28, 49–54]. For translational research, several groups have successfully produced myocardial slices from human hearts [16, 18, 21, 29, 35, 55–58]. Human samples are typically obtained from explanted hearts of patients with end-stage heart failure [5, 20, 27, 35, 47, 48, 59, 60], non-failing donor hearts unsuitable for transplantation [27, 61], and surgical specimens, such as septal myectomies samples [11, 21, 62] or myocardium obtained from left ventricular assist device (LVAD)-supported patients [20, 48, 60].

In 2017, Watson and colleagues published a widely adopted and reproducible protocol for preparing LMS from both small and large animal hearts [2]. A crucial factor is to minimize ischemic damage by obtaining cardiac tissue as rapidly as possible following explantation. Immediately after excision, tissue should be transferred to freshly made, cold (~ 4 °C) cardioplegic solution containing high potassium concentrations and an excitation-contraction uncoupler, typically 2,3-butanedione monoxime (BDM) [2, 4, 63]. This solution slows down enzymatic activity and oxygen consumption, bringing the tissue into a protected, low-energy state that prevents uncontrolled contractions and ionic imbalance. Regarding the transportation and storage, Fischer et al. report that the excised myocardium can be stored in cardioplegic solution for up to 30 h prior to slicing, without reducing the maximum twitch force. However, the effects on other functional or structural parameters remain insufficiently characterized [3, 14]. In addition, Lodrini et al. suggest that slices can even be cryopreserved for subsequent LMS preparation [8].

**Fig. 1:**
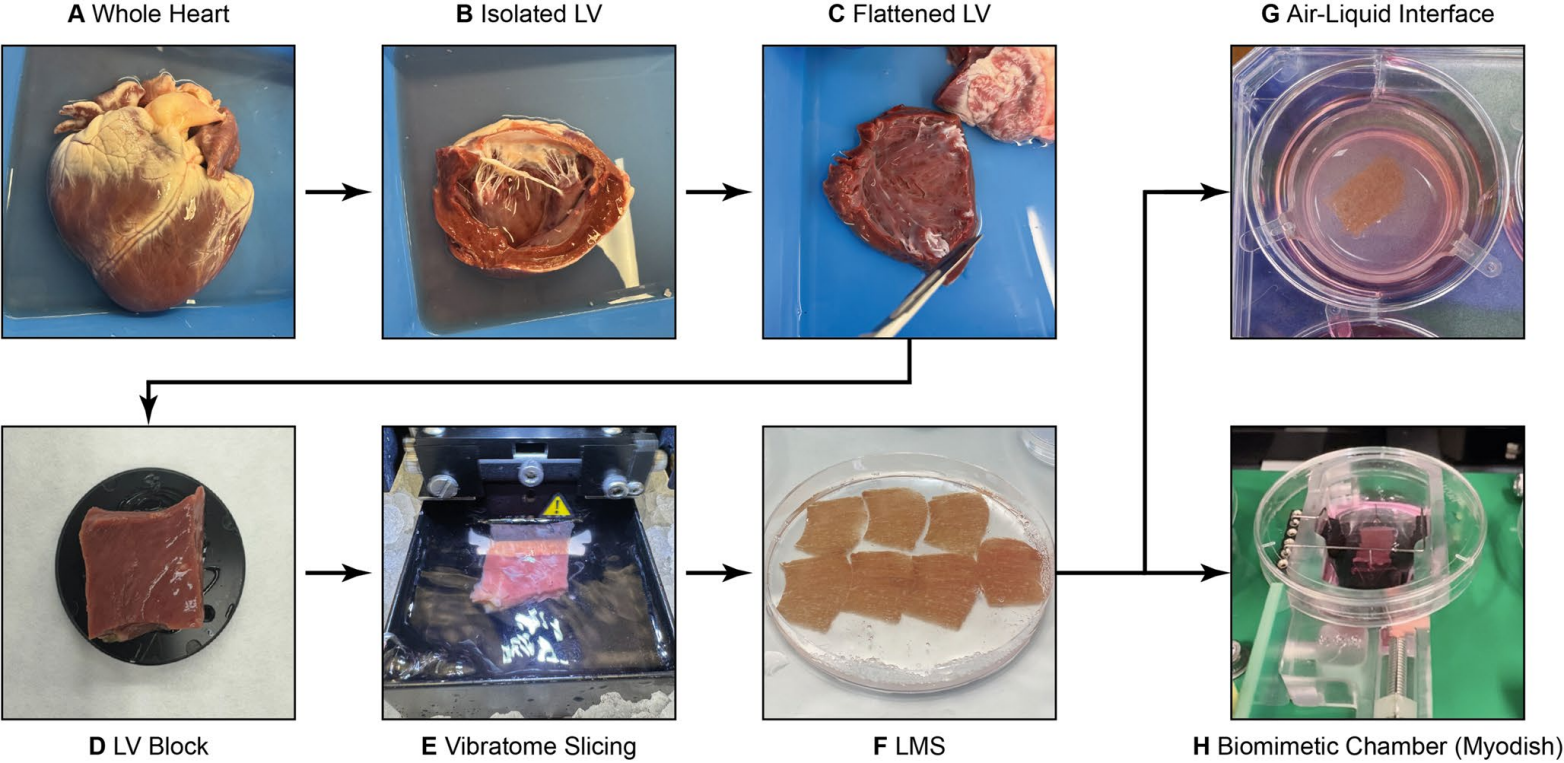
Preparation of LMS from an explanted heart. (**A**) Pig heart obtained from a slaughterhouse. (**B**) The left ventricle is excised. (**C**) The trabeculae and papillary muscles are removed to expose the myocardium and the LV is carefully flattened out. (**D**) Tissue blocks are cut from the flattened left ventricle and glued onto the vibratome holder. (**E**) The tissue block is positioned under the vibratome blade, and vibratome settings (amplitude, speed, and thickness) are set. (**F**) LMS (300 μm) are generated and can be cut into smaller slices if needed. (**G**) Air-liquid interface culturing on a semiporous transwell membrane allows short-term culture for up to 7 days. (**H**) The biomimetic chamber, Myodish (InVitroSys GmbH, Germany), enables electrical and mechanical stimulation and supports long-term culture of LMS for up to 1 month. Images are courtesy of Husetić et al.

For explanted hearts, the left ventricle is isolated, flattened, and an LV tissue block of approximately 1.5 cm^2^ is excised with careful attention to the myocardial fiber direction and alignment. The block is then mounted epicardium-down to the vibratome holder using tissue adhesive [2]. In the case of surgical biopsies, the tissue is already limited in size and typically placed directly on the vibratome holder. In all cases, accurate alignment of the myocardial fiber direction is essential to maximize slice quality, viability and functional performance. To improve stability during the cutting process, some groups embed the tissue blocks in low-melting-point agarose to prevent tissue movement during slicing [59, 64, 65]. Prior to slicing, the vibratome needs to be calibrated by setting the machine’s z-axis vibration to < 0.5 μm to minimize the damage to cardiomyocyte layers [65]. Vibratome settings can vary depending on the tissue source; the most commonly reported settings are a thickness of 300–400 μm, an amplitude of 1–2 mm, and a blade movement speed of 0.03–0.04 mm/s. During slicing, the tissue block should be submerged in ice-cold, oxygenated cutting solution to preserve metabolic stability. A crucial factor is to consider the fiber orientation to minimize damage to the myocardium; therefore, it is recommended to cut along the fibers. For fibrotic or stiff tissue, usually seen in HF patient-derived hearts, reduced cutting speed is recommended [4]. After slicing, LMS can be maintained at room temperature for up to 4 h before transfer to culturing. Several research groups highlight the importance of a post-slicing recovery and washing step to remove residual excitation-uncouplers, especially if functional assays, such as force measurements or electrophysiological recordings, follow directly after slicing [4, 11, 65]. Figure 1 illustrates the preparation workflow of LMS from explanted hearts and the two most common culturing methods. A recent review by Van der Geest et al. reports on the development of various culture methods, discussing their advantages, disadvantages and future directions [10].

## Applications of LMS in Heart Failure Studies

### Heart Failure Modeling

Since the early 2010s, multiple research groups have reported the use of living myocardial slices prepared from human cardiac tissue [48, 63]. In heart failure studies, tissue sources often include explanted hearts from patients with end-stage HF, post-mortem donor hearts and surgical specimens obtained during procedures such as septal myectomies or LVAD implantation. Although these hearts may have underlying diseases that must be considered, their value lies in their ability to more accurately reflect the human myocardial physiological state than conventional animal models, thereby enhancing translational relevance and potentially reducing reliance on in vivo studies. Additionally, using human LMS from HF patients provides the opportunity to model the disease ex vivo, enabling human-specific testing of drug effects, cardiotoxicity, and therapeutic targets, and thereby supporting more translational research [32].

Camelliti and colleagues (2011) were among the first to prepare human slices using left ventricular transmural biopsies derived from end-stage HF patients who underwent heart transplantation or LVAD implantation [48]. Within the same study, for comparison to failing human hearts, the authors included canine slice, commonly used in pharmacological studies. Histological analyses revealed that the slices preserved structural integrity, as demonstrated by longitudinal muscle fiber orientation, gap junctions, and an intact extracellular matrix similar to the native heart structure in vivo. Functional assessment using three potassium channel blockers revealed comparable electrophysiological responses in human and canine slices, which were consistent with data from isolated cardiomyocytes and whole-heart preparations. Therefore, this study provides important proof-of-concept evidence that LMS derived from left ventricular tissue from human hearts are suitable for studying disease mechanisms and drug responses. However, the absence of healthy human control tissue limits direct comparisons between LMS derived from healthy and diseased myocardium.

Building on this foundation, many studies have since included hearts from HF patients to generate LMS for various purposes. Notably, recent investigations have examined whether the human LMS retain patient-specific features [5]. One such study demonstrated significant differences among donor slices, including variations in contractile force and force-frequency relationships. Moreover, pharmacological histories of patients were reflected in corresponding LMS responses. For instance, slices from patients treated with amiodarone showed prolonged action potentials, reduced excitability, and enhanced post-pause potentiation, which is in line with clinical observations [5]. These findings support the concept that LMS can be used as a HF patient-specific ex vivo model and pave the way for pharmacological screening and prevention studies, possibly using LMS to advance personalized medicine.

Together, these studies have provided valuable insights into the use of human slices obtained from HF patients. Many research groups have used them for a variety of applications, including drug testing, cardiotoxicity screening, and evaluation of novel therapeutic drugs and therapies. The following sections will discuss these applications in greater detail.

### Cardiac Ischemia and Injury Modeling

Ischemic heart disease (IHD) is one of the most common causes of HF and is primarily induced by an occlusion of the coronary artery, leading to oxygen and nutrient deprivation [66, 67]. Hence, a wide range of experimental models have been used to study cardiac ischemia in vitro and in vivo, including hypoxic culture systems, nutrient deprivation models or by inducing ischemic damage in animal studies. While in vitro experiments with 2D cell layers can provide valuable insights into cellular responses to hypoxia, they lack multicellular interactions and fail to preserve extracellular matrix integrity. In in vivo rodent studies, myocardial infarction (MI) or ischemia-reperfusion (IR) surgeries are commonly used to model injury, the latter involves permanently (MI) or temporarily (IR) inducing ischemia by ligating the left anterior descending coronary artery, followed by reperfusion to restore blood flow (IR). However, these techniques are highly invasive, technically demanding and subject to substantial variability. Moreover, species-specific differences may limit their predictive value for human disease. Consequently, recent studies have explored the potential of LMS as an ex vivo platform to mimic cardiac ischemia and myocardial damage. This approach addresses the lack of multicellularity in in vitro models, offers greater translational benefit, and potential to reduce animal experiments.

Waleczek and colleagues investigated the effects of prolonged hypoxia (9% O_2_) on rat LMS and characterized functional, molecular, and structural changes [23]. A decline in force generation was already observed after 12 h and became significantly pronounced after 24 h of continuous hypoxic exposure. Hypoxia impaired both systolic and diastolic function and induced significant changes in gene and protein expression related to metabolism and oxidative stress, as well as mitochondrial damage and increased apoptosis. This suggests that LMS recapitulate key features of ischemia-induced myocardial injury.

Pilcher et al. examined whether loss of small nuclear RNA Snord116 protects cardiomyocyte kinetics during ischemic stress [68]. For this purpose, they induced ischemia in slices from Snord116 knockout mice by first perfusing with unoxygenated, glucose-free medium until the bath solution was replaced for about 5 min, then stopping perfusion for 25 min, followed by switching back to oxygenated medium for a 15 min recovery period. This protocol thereby induced ischemia-reperfusion-like injury.

In another study, Abbas and colleagues explored the use of a localized injury model by inducing cryoinjury in rat LMS by exposing part of the slice to a metal probe cooled by submersion in liquid nitrogen [69]. This method caused damage to approximately 30% of the tissue. Injured and non-injured slices were cultured under mechanical load and electrical stimulation. Cryoinjured LMS exhibited impaired contractile function, increased fibrosis, and inflammatory responses, closely resembling post-infarction remodeling observed in vivo*.* Proteomic analysis further revealed an enrichment of ferroptosis-related pathways in cryoinjured LMS. Subsequently, the authors further explored potential therapeutic interventions to reduce the effects of cryoinjury in slices, addressed in a later part of the review.

### Anthracyclines-Induced Cardiotoxicity Studies

Anthracyclines are among the most widely used chemotherapeutic drugs in cancer treatment; however, they are often the leading cause of cardiotoxicity induced by chemotherapy, which can progress to HF if not detected and managed early [70]. Doxorubicin (DOX), in particular, is an effective drug used for chemotherapy; however, up to 23% of treated patients develop signs of HF [71].

Already in 1994, a study by Parrish et al. evaluated whether rat myocardial slices could be a tool for comparative cardiotoxicity research by treating LMS with allylamine (AAM) and doxorubicin (DOX), two well-known drugs known to cause cardiotoxic effects [9]. After 24 h of treatment, both drugs reduced protein synthesis, ATP content and creatine kinase activity, indicating impaired cellular viability. This work represented the first study to demonstrate that LMS could serve as a suitable tool for assessing cardiotoxicity.

In 2020, Miller and colleagues performed a more thorough study, in which they exposed porcine and human LMS to different concentrations of doxorubicin, trastuzumab, and sunitinib, commonly used anthracyclines for cancer treatment, to model cardiotoxicity [47]. Treatment resulted in significant downregulation of cardiac structural and contractile genes, upregulation of oxidative stress responses, electrophysiological remodeling, and decrease in contractility, closely reflecting clinical observations [47]. Transcriptomic analyses of porcine LMS treated with trastuzumab revealed downregulation of genes involved in cardiac muscle contraction, whereas sunitinib treatment led to inhibition of angiogenesis-related genes. These findings demonstrate that exposing LMS to three different cardiotoxins mirrored the known clinical phenotype. While this study was a proof of concept, the authors highlight several important limitations. For instance, the short viability in LMS culture makes them suitable for acute cardiotoxicity studies but not chronic ones, as the cardiotoxins were applied for only 48 h. These constraints underscore the need for improved long-term culture systems or testing additional cardiotoxins in human slices.

Van der Geest et al. further extended this work by applying DOX to human LMS from end-stage HF hearts, which already exhibit high susceptibility to DOX-induced cardiotoxicity. Additionally, the authors explored cardioprotective effects of dexrazoxane, a clinically approved medication used in conjunction with anthracyclines to reduce the risk of clinical HF [27]. Consistent with earlier findings, DOX exposure impaired contractile function and increased structural damage in LMS. After treatment with dexrazoxane, however, contractile function was preserved, and structural damage was prevented, demonstrating that LMS can also be used to evaluate cardioprotective interventions. In line with previous studies, a single-dose exposure to DOX was reported as a limitation, as it does not fully reflect the cyclical administration in clinical practice. This can be resolved through long-term studies using LMS and multiple anthracycline treatments. Nevertheless, based on these studies, LMS have proven to be a suitable 3D model for testing the effect of anthracyclines on heart tissue derived from different species, offering a valuable insight into multicellular effects and changes in cardiac function.

### Therapeutic Drug Testing

As discussed above, several studies have evaluated various pharmacological interventions using animal- and human-derived LMS. An overview of compounds tested on LMS to date is summarized in Table 1. Among others, the drugs tested include cardiotoxic anthracyclines and potassium blockers to capture electrical and contractile changes. Additionally, an increasing number of studies have explored therapeutic drug testing on LMS.

**Table 1:** Pharmacological interventions on LMS

Reference	LMS Source	Pharmacological Interventions	Key Readouts
Parrish et al. [9]	Rat LMS	Allylamine Doxorubicin	*Molecular*: Protein synthesis, ATP content, cytosolic enzyme release, oxidative stress (lipid peroxidation)
Bussek et al. [44]	Guinea pig LMS Rat LMS	E4031 Nifedipine Risperidone	*Molecular*: Histology (2-photon microscopy) *Functional*: Electrophysiology (AP, FP, APD, FPD, CV)
Camelliti et al. [48]	Human LMS from left ventricular transmural biopsies from end-stage HF patients with DCM or from LVAD implantation Canine LMS	E4031 Chromanol 293B 4-aminopyridine	*Molecular*: Structural morphology and viability, metabolic viability (ATP/ADP, PCr/Cr) *Functional*: Electrophysiology (AP, FP, APD, FPD, CV)
Brandenburger et al. [11]	Human LMS patients undergoing septal myectomy	Ion-channel modulators ($I_{Kr}$blockers, ATP‐dependent K+ channel modulators)	*Molecular*: Structural morphology and viability, ion channel expression *Functional*: Electrophysiology (AP, APD, excitability, β-adrenergic response), contractility
Thomas et al. [59]	Human LMS from LV free wall from hearts removed at the time of transplant, or from donors whose hearts were not transplanted	Isoproterenol Propranolol A61603 Phenylephrine	*Molecular*: Morphology, diffusion and viability, sarcomere length measurements *Functional*: Contractile force; adrenergic signaling (pPLN, pERK);
Perbellini et al. [35]	Human LMS from explanted hearts of end-stage heart-failure patients Canine LMS	TGFβ, Ang II, VEGFα	*Molecular*: Second harmonic generation (SHG) imaging for visualizing collagen content, live/dead assay, sarcomere length
Miller et al. [47]	Porcine LMS Human LMS from donor hearts	Trastuzumab Sunitinib Doxorubicin	*Molecular: Transcriptional profiling (bulk-RNA seq), tissue viability and structure, calcium-transient assessment *Functional*: Contractility, excitation (optical mapping)
Amesz et al. [60]	Human LMS from end-stage heart-failure patients or from LVAD surgery	Omecamtiv mecarbil Dobutamine	*Functional*: Contractility, contraction force, FRP, FFR, TTP, TTR
Boukhalfa et al. [13]	Canine LMS	Doxorubicin Rapamycin	*Molecular*: Viability, autophagic flux, apoptosis
Amesz et al. [20]	Human LMS from heart transplantation or LVAD surgery	Empagliflozin	*Functional*: Contractility, TTP, TTR
Schmidt et al. [72]	Human LMS from end-stage heart-failure patients	Empagliflozin Dapagliflozin	*Molecular: Transcriptional profiling (single-nucleus RNA seq)
Abbas et al. [69]	Rat LMS Human LMS from surgical specimens (not specified)	Ferrostatin-1 Dexrazoxane	*Molecular*: Viability, cardiac remodeling, proteomic profiling *Functional*: Contractility
Amesz et al. [21]	Human LMS from left ventricular outflow tract of patients with HCM undergoing left ventricular septal myectomy	Mavacamten Verapamil	*Functional*: Contractility, temporal/kinetic contraction metric, FRP, FRR electrophysiology
Van der Geest et al. [27]	Human LMS from left ventricle free wall of patients with end-stage HF or immediate postmortem examination	Doxorubicin Dexrazoxane	*Molecular: Calcium transient, glucose consumption, structural integrity, transcriptomic profiling (bulk-RNA seq) *Functional*: Contractility, excitability
Krammer et al. [26]	Human LMS from patients with aortic stenosis and patients with end-stage HF with reduced ejection fraction undergoing heart transplantation	Semaglutide	*Functional*: Contractility
Kiselev et al. [62]	Porcine LMSHuman LMS from myectomy from patients with HOCM	Mavacamten	*Molecular: Transcriptional profiling (bulk RNA-seq)*Functional*: Contractility

Overview of pharmacological interventions tested in LMS, including cardiac tissue source, chemical compounds or therapeutic drugs used, and key experimental readouts. Molecular readouts include gene and protein expression analyses, histology, assessment of fibrosis and extracellular matrix composition, inflammatory and cell death markers, measurements of energy metabolism, specifically ATP/ADP and phosphocreatine/creatine (PCr/Cr) ratios, and markers of oxidative stress. Studies using high-dimensional molecular technologies, such as bulk or single-cell/single-nucleus transcriptomics or proteomics, are indicated by an asterisk (*). Functional readouts refer to myocardial performance and electrophysiology, including contractile performance and force generation, calcium handling, and electrical conduction properties. Electrophysiological parameters include action potentials (AP), field potentials (FP), action potential duration (APD), field potential duration (FPD), conduction velocity (CV), force-frequency relationship (FFR), functional refractory period (FRP), time to peak contraction (TTP), and time to relaxation (TTR)

One study combined an injury model with therapeutic drug testing by pharmacologically inhibiting ferroptosis in cryoinjured rat LMS using ferrostatin [69]. Ferrostatin-1 (Fer-1) treatment for 24h led to repression of fibrotic processes, improved contractile properties, and increased tissue viability in an acute injury model in rat LMS.

To assess the relevance of ferroptosis inhibition in chronic HF, the same group subsequently treated LMS derived from failing human hearts with ferrostatin for three days [69]. The initial results were similar to those in the acute injury model. Fer‑1 treatment significantly increased the contractile force compared to controls, indicating improved contractile function, while contraction and relaxation times remained unchanged. In both models, treatment improved tissue viability. In contrast to rat LMS, which showed a reduction in fibrotic processes, human LMS did not show a significant change in expression of fibroblast genes or interstitial collagen I, possibly reflecting preexisting pathological remodeling of the tissue. Nevertheless, these findings demonstrate that LMS can be used to evaluate cardioprotective interventions in both acute and chronic disease contexts.

Another study by Amesz et al. explored the effect of omecamtiv mecarbil (OM), a selective cardiac myosin activator, on human LMS from patients undergoing heart transplantation or LVAD implantation [60]. OM has been proposed as a novel therapy for HF patients, as it is able to increase cardiomyocyte contractility in pre-clinical in vitro and in vivo studies. In this study, human LMS were exposed to OM and compared to dobutamine, a potent β1-adrenergic receptor agonist known to increase myocardial contractility [60]. Both compounds increased maximal contractile force and prolonged systolic duration, indicating improved cardiac function. However, OM alone significantly impaired relaxation, especially at higher pacing frequencies and shortened the functional refractory period (FRP). The authors acknowledge both the beneficial and adverse effects of OM that should be further explored with different dosing. This study provided interesting and critical insights into the impact of OM on the heart and, for the first time, used LMS to study this potentially therapeutic drug.

Sodium-glucose cotransporter 2 inhibitors (SGLT2i) have also been investigated using LMS. Although SGLT2 is minimally expressed in the heart, SGLT2i have shown cardioprotective effects in clinical studies, potentially by decreasing oxidative stress, fibrosis, and inflammatory signaling and promoting a metabolic shift that improves myocardial energy supply. Two studies have explored the effect of empagliflozin (EMPA), a common SGLT2i, in slices. One study used LMS from end-stage HF patients to investigate the acute biomechanical effect of EMPA on slices under mechanical preload and electrical stimulation. While treatment with EMPA did not affect the maximum contraction force of the slices, it significantly increased contraction duration, suggesting that the drug can improve cardiac time intervals [20]. However, the study mainly focused on mechanical force measurements and did not assess changes in molecular mechanisms.

Molecular and cell-specific changes were addressed in a study by another group that treated human LMS with EMPA or dapagliflozin (DAPA) for 24 h, followed by single-nucleus RNA sequencing [72]. Both SGLT2is had effects mostly in three larger cell populations: endothelial cells, mural cells, and fibroblasts, with EMPA showing more potent effects overall. The findings suggest that SGLT2 inhibitors have cell-type-specific effects, including modulation of extracellular matrix genes in fibroblasts and mural cells and changes in calcium signaling and mitochondrial homeostasis-related genes in cardiomyocytes. These transcriptomic changes were consistent with clinical observations in HF patients receiving SGLT2i therapy. Despite these advances, the authors acknowledge several limitations, including the lack of a healthy control tissue, which would provide a baseline for comparing the SGLT2i effects, and the absence of functional measurements. These aspects may be addressed in future studies, for example, by incorporating an electrical stimulation protocol. Moreover, this work also highlights the utility of LMS as a platform for testing the therapeutic benefits of drugs at single-cell resolution and with transcriptomic profiling, which has been challenging to achieve with other models.

### Gene Therapy and Pro-Regenerative Therapies

Beyond conventional drug testing, LMS have recently been used to study cell-free therapeutic approaches, which aim to promote cardiac repair and have emerged as promising next-generation strategies for heart regeneration [73]. Gene therapy enables targeted delivery of viral or non-viral vectors carrying therapeutic genes into cardiac tissue and has progressed towards clinical applications. Among available delivery systems, adeno-associated viral (AAV) vectors have become the standard for in vitro and in vivo gene therapy and a potential tool for treating HF, due to their stable expression of therapeutic genes in the heart [74]. Nevertheless, reliable preclinical platforms are required for evaluating gene therapy efficacy and safety prior to clinical translation. Due to their physiological relevance and multicellularity, LMS have recently been employed in several studies as a platform for AAV-mediated gene transfer and for testing pro-regenerative therapies.

The first study that explored the use of LMS as a platform for cardiac gene therapy was reported by Liu et al. in 2020. Using murine myocardial slices, the authors showed efficient AAV-mediated gene transfer while maintaining the viability of slices for 5 days [32]. None of the tested serotypes (AAV1, AAV2, AAV6 and AAV8) induced detectable cytotoxicity in LMS, and AAV6 exhibited the highest transduction efficiency, as determined by GFP-positive cell quantification. Notably, the proportion of GFP-positive cells was eight times higher in cardiomyocytes compared to GFP-positive fibroblasts. However, AAV9, a serotype commonly used in in vivo studies, was not evaluated. Moreover, the authors do not report whether the AAV6 serotype targets endothelial and smooth muscle cells in the slices.

To explore the use of AAV9 vectors, a subsequent study used rat-derived LMS and compared transduction with either AAV6-GFP or AAV9-GFP at different multiplicity of infection (MOIs). Consistent with earlier findings, they reported that AAV6 achieved significantly higher transduction efficiency in cardiomyocytes compared to AAV9. Importantly, neither vector affected the contractility of active force. The authors performed the same experiment with human LMS derived from explanted hearts with dilated cardiomyopathy and obtained similar results. This study, amongst those mentioned previously, demonstrates that LMS from several species are suitable for gene therapy using AAVs, with AAV6 being the serotype with the highest transduction efficiency. This means that LMS can be used to deliver pro-regenerative targets to heart tissue and preserve contractile function and structural integrity. Another study employed human atrial myocardial slices, together with hiPSC-CMs and other in vitro models, and further confirmed the superior transduction capacity of AAV6 in slices compared to AAV9. This result was consistent with in vivo intramyocardial delivery data [75].

To establish whether LMS can reproduce regenerative responses observed in vivo, Caliandro and colleagues conducted two studies to evaluate pro-regenerative gene targets using AAV-mediated delivery to LMS [76]. In their initial work, they used pig-derived LMS to test three pro-regenerative targets: zinc finger E-box-binding homeobox 2 (ZEB2), thymosin beta-4 (TMSB4), and prothymosin alpha (PTMA). The latter two were delivered in combination as their secretion is regulated by ZEB2 [12]. Results from porcine LMS transduced with AAV6 or AAV9 were compared with in vivo AAV9-mediated delivery in mice. The authors report that delivery of ZEB2 and TMSB4 + PTMA enhanced an angiogenic response in both models based on transcriptomic data and histological analysis showing increased PECAM1-positive areas. Thereby, they demonstrate that porcine LMS can reliably recapitulate in vivo outcomes. Furthermore, while both serotypes effectively transduce cardiomyocytes, AAV6 delivery showed a broader, mosaic-like GFP expression pattern, suggesting more uniform transduction efficiency in LMS cardiomyocytes.

Building on these findings, the authors subsequently investigated whether AAV6-mediated delivery of ZEB2 could lead to similar effects in adult human LMS derived from donor hearts [61]. Consequently, treatment with ZEB2 increased the vessel area in the slice, confirming its pro-angiogenic effect in human LMS. Moreover, transcriptomic analyses further revealed activation of pathways associated with cardiomyocyte dedifferentiation and cellular plasticity. The authors concluded that ZEB2 promotes reprogramming of adult human cardiomyocytes toward a more dedifferentiated phenotype, indicating that ZEB2 can potentially promote cardiac repair in human heart tissue.

Collectively, these studies demonstrate that LMS derived from both pig and human hearts can faithfully reproduce regenerative processes observed in vivo. Thereby, they proved that LMS can be used as a physiologically relevant platform for testing pro-regenerative therapies, providing a reliable alternative to animal experiments.

### Device Therapy in Failing Human LMS

Implantable devices, such as pacemakers or left ventricular assist devices (LVAD), represent a crucial aspect of HF management by supporting residual cardiac function and improving clinical outcomes. The development and testing of such devices is essential and requires a physiologically relevant models that resemble human myocardial tissue. In this context, LMS allow the unique opportunity to model device-based interventions under controlled conditions where mechanical loading and electrical stimulation can be applied. To date, relatively few studies have explored the use of LMS for device therapy research. One study has explored the use of LMS specifically for testing device therapy. Cardiac contractility modulation (CCM) is a new device-based therapy for HF that delivers high-voltage, long-duration electrical pulses to the myocardium during the refractory period, aiming to enhance myocardial contractility not by creating new contractions but by increasing intracellular calcium levels during systole [24]. Previous studies relied on 3D engineered heart tissues and simplified in vitro models to test how the CCM can impact cardiomyocyte contractility. However, these models typically do not include mechanical loading. To overcome these limitations, a recent study was conducted using LMS that were mechanically loaded in a biomimetic chamber, a culturing system for LMS previously mentioned [24]. The goal of the study was to assess the acute effect of CCM on the biomechanical properties of LMS from end-stage HF-derived patients. CCM parameters were mimicked in the biomimetic culturing system. Interestingly, acute CCM stimulation significantly increased maximum contractile force without affecting contraction duration, indicating improved mechanical performance. These findings provided proof-of-concept evidence that LMS derived from failed human hearts serve as a functional platform for assessing device-based therapies. Nevertheless, several limitations should be considered. As in most studies with end-stage HF, the absence of a non-failing healthy control group restricted comparative analyses. Moreover, the study focused on an acute biomechanical effect, demonstrating the impact of CCM over a short time only, and thereby paving the way for future research to investigate the chronic effects of CCM in slices.

## Conclusions

Living myocardial slices have become a promising tool in cardiac translational research, bridging the gap between conventional in vitro and in vivo models by more closely replicating the complexity and function of the human myocardium. The application of LMS has enabled a more physiologically relevant model of HF and its underlying diseases, mimics device therapy, and supports preclinical testing of pharmacological and gene-based interventions. As with any experimental model, important challenges remain. These include limited tissue availability, donor variability, and the need for standardized preparation and culture protocols. Looking ahead, standardization of LMS preparation and the experimental protocols will be essential, as current variability in slicing techniques and culturing conditions limits reproducibility and comparability across studies. The implementation of standardized quality control criteria for slice viability and functionality could help address this issue. Moreover, further optimization of culture systems will be essential in order to extend culture duration and better preserve structural and functional integrity over time. Overall, given the rising number of studies, LMS have become a widely recognized model in cardiac research, a tool for understanding pathophysiology, and a platform with the potential to accelerate the development of effective therapies for heart failure, including more personalized approaches.

## Key References

- Watson SA, Scigliano M, Bardi I, Ascione R, Terracciano CM, Perbellini F. Preparation of viable adult ventricular myocardial slices from large and small mammals. Nat Protoc. 2017;12(12):2623–2639.
  - ○ This protocol paper describes a standardized method for the preparation of viable ventricular myocardial slices from small and large animals.
- Fischer C, Milting H, Fein E, Reiser E, Lu K, Seidel T, et al. Long-term functional and structural preservation of precision-cut human myocardium under continuous electromechanical stimulation in vitro. Nat Commun. 2019;10(1):117.
  - ○ This study reports the use of a commercially available and widely used culturing system for long-term maintenance of living myocardial slices under electromechanical stimulation.
- Camelliti P, Al-Saud SA, Smolenski RT, Al-Ayoubi S, Bussek A, Wettwer E, Banner NR, Bowles CT, Yacoub MH, Terracciano CM. Adult human heart slices are a multicellular system suitable for electrophysiological and pharmacological studies. J Mol Cell Cardiol. 2011 Sep;51(3):390–398.
  - ○ This paper demonstrates that adult human heart slices preserve multicellular architecture of the heart and are suitable for electrophysiological and pharmacological studies.
- van der Geest JSA, Kelters IR, Arends B, van Ham WB, Benavente ED, Lapre TA, et al. Dexrazoxane protects against doxorubicin-induced cardiotoxicity in susceptible human living myocardial slices: A proof-of-concept study. Br J Pharmacol. 2025;182(18):4262–4280.
  - ○ This study shows that human living myocardial slices reproduce clinically relevant doxorubicin-induced cardiotoxicity and can be used to assess cardioprotective interventions.
- Caliandro R, Husetić A, Ligtermoet ML, Boender AR, Zentilin L, Boink GJJ, et al. Living myocardial slices as a model for testing cardiac pro-reparative gene therapies. Mol Ther. 2025;33(7):2990–2996.
  - ○ This manuscript highlights living myocardial slices as a suitable model for evaluating pro-regenerative cardiac therapies using viral-mediated gene delivery.

## Data Availability

No datasets were generated or analysed during the current study.

## References

[1] Savarese G, Becher PM, Lund LH, Seferovic P, Rosano GMC, Coats AJS. Global burden of heart failure: a comprehensive and updated review of epidemiology. Cardiovasc Res. 2023;118(17):3272–87. 35150240 10.1093/cvr/cvac013

[2] Watson SA, Scigliano M, Bardi I, Ascione R, Terracciano CM, Perbellini F. Preparation of viable adult ventricular myocardial slices from large and small mammals. Nat Protoc. 2017;12(12):2623–39. 29189769 10.1038/nprot.2017.139

[3] Perbellini F, Thum T. Living myocardial slices: a novel multicellular model for cardiac translational research. Eur Heart J. 2020;41(25):2405–8. 31711161 10.1093/eurheartj/ehz779 PMC7327529

[4] Watson SA, Dendorfer A, Thum T, Perbellini F. A practical guide for investigating cardiac physiology using living myocardial slices. Basic Res Cardiol. 2020;115(6):61. 32914360 10.1007/s00395-020-00822-y PMC7496048

[5] van der Geest JSA, van Ham WB, Benavente ED, El Amrani M, Mokhles MM, Oerlemans M, et al. Living myocardial slices retain patient-specific features: Insights into etiology and therapeutic history. JHLT Open. 2025;9:100345. 40799359 10.1016/j.jhlto.2025.100345 PMC12341583

[6] Pincus MH. Effect of pitressin and pitocin on oxygen consumption of excised tissue. P Soc Exp Biol Med. 1933;30(8):1171–4.

[7] Smith RE. Comparative evaluation of 2 instruments and procedures to cut nonfrozen sections. J Histochem Cytochem. 1970;18(8):590–1. 5449984 10.1177/18.8.590

[8] Lodrini AM, Groeneveld EJ, Palmen M, Hjortnaes J, Smits AM, Goumans MJ. Cryopreservation of Human Adult Ventricular Tissue for the Preparation of Viable Myocardial Slices. Curr Protoc. 2024;4(12):e70068. 39625241 10.1002/cpz1.70068 PMC11613814

[9] Parrish AR, Dorr RT, Gandolfi AJ, Brendel K. Adult rat myocardial slices: A tool for studies of comparative cardiotoxicity. Toxicol Vitro. 1994;8(6):1233–7. 10.1016/0887-2333(94)90114-7 20693093

[10] van der Geest JSA, de Boer TP, Terracciano CM, Thum T, Dendorfer A, Doevendans PA, et al. Living myocardial slices: walking the path towards standardization. Cardiovasc Res. 2025;121(7):1011–23. 40354127 10.1093/cvr/cvaf079 PMC12236068

[11] Brandenburger M, Wenzel J, Bogdan R, Richardt D, Nguemo F, Reppel M, et al. Organotypic slice culture from human adult ventricular myocardium. Cardiovasc Res. 2012;93(1):50–9. 21972180 10.1093/cvr/cvr259

[12] Caliandro R, Husetić A, Ligtermoet ML, Boender AR, Zentilin L, Boink GJJ, Giacca M, Gladka MM. Living myocardial slices as a model for testing cardiac pro-reparative gene therapies. Mol Ther. 2025 Jul 2;33(7):2990-2996. 40143546 10.1016/j.ymthe.2025.03.033 PMC12266023

[13] Boukhalfa A, Robinson SR, Meola DM, Robinson NA, Ling LA, LaMastro JN, et al. Using cultured canine cardiac slices to model the autophagic flux with doxorubicin. PLoS ONE. 2023;18(3):e0282859. 36928870 10.1371/journal.pone.0282859 PMC10019679

[14] Fischer C, Milting H, Fein E, Reiser E, Lu K, Seidel T, et al. Long-term functional and structural preservation of precision-cut human myocardium under continuous electromechanical stimulation in vitro. Nat Commun. 2019;10(1):117. 30631059 10.1038/s41467-018-08003-1 PMC6328583

[15] Abu-Khousa M, Fiegle DJ, Sommer ST, Minabari G, Milting H, Heim C, et al. The Degree of t-System Remodeling Predicts Negative Force-Frequency Relationship and Prolonged Relaxation Time in Failing Human Myocardium. Front Physiol. 2020;11:182. 32231589 10.3389/fphys.2020.00182 PMC7083140

[16] Bojkova D, Wagner JUG, Shumliakivska M, Aslan GS, Saleem U, Hansen A, et al. SARS-CoV-2 infects and induces cytotoxic effects in human cardiomyocytes. Cardiovasc Res. 2020;116(14):2207–15. 32966582 10.1093/cvr/cvaa267 PMC7543363

[17] Moretti A, Fonteyne L, Giesert F, Hoppmann P, Meier AB, Bozoglu T, et al. Somatic gene editing ameliorates skeletal and cardiac muscle failure in pig and human models of Duchenne muscular dystrophy. Nat Med. 2020;26(2):207–14. 31988462 10.1038/s41591-019-0738-2 PMC7212064

[18] Esfandyari D, Idrissou BMG, Hennis K, Avramopoulos P, Dueck A, El-Battrawy I, et al. MicroRNA-365 regulates human cardiac action potential duration. Nat Commun. 2022;13(1):220. 35017523 10.1038/s41467-021-27856-7 PMC8752767

[19] Hamers J, Sen P, Merkus D, Seidel T, Lu K, Dendorfer A. Preparation of human myocardial tissue for long-term cultivation. J Vis Exp. 2022;(184):e63964. 10.3791/63964 35723462

[20] Amesz JH, Langmuur SJJ, Epskamp N, Bogers A, de Groot NMS, Manintveld OC, et al. Acute Biomechanical Effects of Empagliflozin on Living Isolated Human Heart Failure Myocardium. Cardiovasc Drugs Ther. 2024;38(4):659–66. 36780068 10.1007/s10557-023-07434-3 PMC11266265

[21] Amesz JH, Langmuur SJJ, Zhang L, Manintveld OC, Schinkel AFL, de Jong PL, et al. Biomechanical response of ultrathin slices of hypertrophic cardiomyopathy tissue to myosin modulator mavacamten. Biomed Pharmacother. 2024;170:116036. 38134635 10.1016/j.biopha.2023.116036

[22] Sun Z, Lu K, Kamla C, Kameritsch P, Seidel T, Dendorfer A. Synchronous force and Ca(2+) measurements for repeated characterization of excitation-contraction coupling in human myocardium. Commun Biol. 2024;7(1):220. 38388802 10.1038/s42003-024-05886-3 PMC10884022

[23] Waleczek FJG, Cipriano G, Haas JA, Garg A, Pfanne A, Just A, et al. Prolonged hypoxia in rat living myocardial slices affects function, expression, and structure. Int J Mol Sci. 2024;26(1):218. 39796086 10.3390/ijms26010218 PMC11720517

[24] Bierhuizen MFA, Amesz JH, Langmuur SJJ, Lam B, Knops P, Veen KM, et al. Acute biomechanical effects of cardiac contractility modulation in living myocardial slices from end-stage heart failure patients. Bioeng (Basel). 2025;12(2):174. 10.3390/bioengineering12020174 PMC11851609 40001693

[25] Chan AS, Greiner J, Marschhauser L, Brennan TA, Perez-Feliz S, Agrawal A, et al. Spatiotemporal dynamics of the cardioimmune niche during lesion repair. Nat Cardiovasc Res. 2025;4(11):1550–72. 41184578 10.1038/s44161-025-00739-6 PMC12611762

[26] Krammer T, Baier MJ, Hegner P, Zschiedrich T, Lukas D, Wolf M, et al. Cardioprotective effects of semaglutide on isolated human ventricular myocardium. Eur J Heart Fail. 2025;27(7):1315–25. 40107718 10.1002/ejhf.3644 PMC12370581

[27] van der Geest JSA, Kelters IR, Arends B, van Ham WB, Benavente ED, Lapre TA, et al. Dexrazoxane protects against doxorubicin-induced cardiotoxicity in susceptible human living myocardial slices: A proof-of-concept study. Br J Pharmacol. 2025;182(18):4262–80. 40437840 10.1111/bph.70085

[28] Maselli D, Matos RS, Johnson RD, Martella D, Caprettini V, Chiappini C, et al. Porcine Organotypic Epicardial Slice Protocol: A Tool for the Study of Epicardium in Cardiovascular Research. Front Cardiovasc Med. 2022;9:920013. 35924218 10.3389/fcvm.2022.920013 PMC9339655

[29] Amesz JH, Zhang L, Everts BR, De Groot NMS, Taverne Y. Living myocardial slices: Advancing arrhythmia research. Front Physiol. 2023;14:1076261. 36711023 10.3389/fphys.2023.1076261 PMC9880234

[30] Cao-Ehlker X, Fischer C, Lu K, Bruegmann T, Sasse P, Dendorfer A, et al. Optimized conditions for the long-term maintenance of precision-cut murine myocardium in biomimetic tissue culture. Bioeng (Basel). 2023;10(2):171. 10.3390/bioengineering10020171 PMC9952453 36829664

[31] Mihai MC, Popa MA, Suica VI, Antohe F, Jackson EK, Leeners B, et al. Proteomic analysis of estrogen-mediated enhancement of mesenchymal stem cell-induced angiogenesis *in vivo*. Cells. 2021;10(9):2181. 34571830 10.3390/cells10092181 PMC8468955

[32] Liu Z, Klose K, Neuber S, Jiang M, Gossen M, Stamm C. Comparative analysis of adeno-associated virus serotypes for gene transfer in organotypic heart slices. J Transl Med. 2020;18(1):437. 33208161 10.1186/s12967-020-02605-4 PMC7673099

[33] He S, Wen Q, O’Shea C, Mu UMR, Kou K, Grassam-Rowe A, et al. A protocol for transverse cardiac slicing and optical mapping in murine heart. Front Physiol. 2019;10:755. 31293436 10.3389/fphys.2019.00755 PMC6603341

[34] Peinkofer G, Hescheler J, Halbach M. Murine short axis ventricular heart slices for electrophysiological studies. J Vis Exp. 2017;(124):55725. 10.3791/55725 PMC5608244 28605368

[35] Perbellini F, Watson SA, Scigliano M, Alayoubi S, Tkach S, Bardi I, et al. Investigation of cardiac fibroblasts using myocardial slices. Cardiovasc Res. 2018;114(1):77–89. 29016704 10.1093/cvr/cvx152 PMC5852538

[36] Dries E, Bardi I, Nunez-Toldra R, Meijlink B, Terracciano CM. CaMKII inhibition reduces arrhythmogenic Ca2 + events in subendocardial cryoinjured rat living myocardial slices. J Gen Physiol. 2021;153(6):e202012737. 33956073 10.1085/jgp.202012737 PMC8105719

[37] Song X, Wang L, Liu M, Pan R, Song J, Kong J. Atractylenolide II ameliorates myocardial fibrosis and oxidative stress in spontaneous hypertension rats. Technol Health Care. 2024;32(1):131–42. 37483026 10.3233/THC-220601

[38] Nunez-Toldra R, Del Canizo A, Secco I, Nicastro L, Giacca M, Terracciano CM. Living myocardial slices for the study of nucleic acid-based therapies. Front Bioeng Biotechnol. 2023;11:1275945. 37941724 10.3389/fbioe.2023.1275945 PMC10628718

[39] Zabielska-Kaczorowska MA, Bogucka AE, Macur K, Czaplewska P, Watson SA, Perbellini F, et al. Label-free quantitative SWATH-MS proteomic analysis of adult myocardial slices in vitro after biomimetic electromechanical stimulation. Sci Rep. 2022;12(1):16533. 36192624 10.1038/s41598-022-20494-z PMC9529937

[40] Waleczek FJG, Sansonetti M, Xiao K, Jung M, Mitzka S, Dendorfer A, et al. Chemical and mechanical activation of resident cardiac macrophages in the living myocardial slice ex vivo model. Basic Res Cardiol. 2022;117(1):63. 36449104 10.1007/s00395-022-00971-2 PMC9712328

[41] Pitoulis FG, Hasan W, Papadaki M, Clavere NG, Perbellini F, Harding SE, et al. Intact myocardial preparations reveal intrinsic transmural heterogeneity in cardiac mechanics. J Mol Cell Cardiol. 2020;141:11–6. 32201175 10.1016/j.yjmcc.2020.03.007 PMC7246333

[42] Watson SA, Duff J, Bardi I, Zabielska M, Atanur SS, Jabbour RJ, et al. Biomimetic electromechanical stimulation to maintain adult myocardial slices in vitro. Nat Commun. 2019;10(1):2168. 31092830 10.1038/s41467-019-10175-3 PMC6520377

[43] Wang K, Lee P, Mirams GR, Sarathchandra P, Borg TK, Gavaghan DJ, et al. Cardiac tissue slices: preparation, handling, and successful optical mapping. Am J Physiol Heart Circ Physiol. 2015;308(9):H1112–25. 25595366 10.1152/ajpheart.00556.2014 PMC4551126

[44] Bussek A, Wettwer E, Christ T, Lohmann H, Camelliti P, Ravens U. Tissue slices from adult mammalian hearts as a model for pharmacological drug testing. Cell Physiol Biochem. 2009;24(5–6):527–36. 19910693 10.1159/000257528

[45] Bussek A, Schmidt M, Bauriedl J, Ravens U, Wettwer E, Lohmann H. Cardiac tissue slices with prolonged survival for in vitro drug safety screening. J Pharmacol Toxicol Methods. 2012;66(2):145–51. 22245702 10.1016/j.vascn.2011.12.002

[46] Pfeuffer AM, Kupfer LK, Shankar TS, Drakos SG, Volk T, Seidel T. Ryanodine receptor staining identifies viable cardiomyocytes in human and rabbit cardiac tissue slices. Int J Mol Sci. 2023;24(17):13514. 37686327 10.3390/ijms241713514 PMC10488113

[47] Miller JM, Meki MH, Ou Q, George SA, Gams A, Abouleisa RRE, et al. Heart slice culture system reliably demonstrates clinical drug-related cardiotoxicity. Toxicol Appl Pharmacol. 2020;406:115213. 32877659 10.1016/j.taap.2020.115213 PMC7554180

[48] Camelliti P, Al-Saud SA, Smolenski RT, Al-Ayoubi S, Bussek A, Wettwer E, et al. Adult human heart slices are a multicellular system suitable for electrophysiological and pharmacological studies. J Mol Cell Cardiol. 2011;51(3):390–8. 21740909 10.1016/j.yjmcc.2011.06.018

[49] Ou Q, Jacobson Z, Abouleisa RRE, Tang XL, Hindi SM, Kumar A, et al. Physiological Biomimetic Culture System for Pig and Human Heart Slices. Circ Res. 2019;125(6):628–42. 31310161 10.1161/CIRCRESAHA.119.314996 PMC6715512

[50] Wu Q, Ross AJ, Ipek T, Thompson GH, Johnson RD, Wu C, et al. Hydroxychloroquine and azithromycin alter the contractility of living porcine heart slices. Front Pharmacol. 2023;14:1127388. 37214466 10.3389/fphar.2023.1127388 PMC10196358

[51] Shi R, Reichardt M, Fiegle DJ, Kupfer LK, Czajka T, Sun Z, et al. Contractility measurements for cardiotoxicity screening with ventricular myocardial slices of pigs. Cardiovasc Res. 2023;119(14):2469–81. 37934066 10.1093/cvr/cvad141 PMC10651213

[52] Wang B, Shah M, Williams LN, de Jongh Curry AL, Hong Y, Zhang G, et al. Acellular Myocardial Scaffolds and Slices Fabrication, and Method for Applying Mechanical and Electrical Simulation to Tissue Construct. Methods Mol Biol. 2022;2485:55–70. 35618898 10.1007/978-1-0716-2261-2_4 PMC9811994

[53] Abdeltawab H, Khalifa F, Hammouda K, Miller JM, Meki MM, Ou Q, et al. Artificial Intelligence Based Framework to Quantify the Cardiomyocyte Structural Integrity in Heart Slices. Cardiovasc Eng Technol. 2022;13(1):170–80. 34402037 10.1007/s13239-021-00571-6 PMC8847536

[54] Miller JM, Meki MH, Elnakib A, Ou Q, Abouleisa RRE, Tang XL, et al. Biomimetic cardiac tissue culture model (CTCM) to emulate cardiac physiology and pathophysiology ex vivo. Commun Biol. 2022;5(1):934. 36085302 10.1038/s42003-022-03919-3 PMC9463130

[55] Fusco-Allison G, Li DK, Hunter B, Jackson D, Bannon PG, Lal S, et al. Optimizing the discovery and assessment of therapeutic targets in heart failure with preserved ejection fraction. ESC Heart Fail. 2021;8(5):3643–55. 34342166 10.1002/ehf2.13504 PMC8497375

[56] Qiao Y, Dong Q, Li B, Obaid S, Miccile C, Yin RT, et al. Multiparametric slice culture platform for the investigation of human cardiac tissue physiology. Prog Biophys Mol Biol. 2019;144:139–50. 29960680 10.1016/j.pbiomolbio.2018.06.001

[57] George SA, Brennan JA, Efimov IR. Preclinical cardiac electrophysiology assessment by dual voltage and calcium optical mapping of human organotypic cardiac slices. J Vis Exp. 2020;(160):e60781. 10.3791/60781 32628156

[58] Chowdhury RA, Tzortzis KN, Dupont E, Selvadurai S, Perbellini F, Cantwell CD, et al. Concurrent micro- to macro-cardiac electrophysiology in myocyte cultures and human heart slices. Sci Rep. 2018;8(1):6947. 29720607 10.1038/s41598-018-25170-9 PMC5932023

[59] Thomas RC, Singh A, Cowley P, Myagmar BE, Montgomery MD, Swigart PM, et al. A Myocardial Slice Culture Model Reveals Alpha-1A-Adrenergic Receptor Signaling in the Human Heart. JACC Basic Transl Sci. 2016;1(3):155–67. 27453955 10.1016/j.jacbts.2016.03.005 PMC4955869

[60] Amesz JH, Langmuur SJJ, Bierhuizen MFA, de Groot NMS, Manintveld OC, Taverne Y. Omecamtiv mecarbil in precision-cut living heart failure slices: A story of a double-edged sword. J Mol Cell Cardiol Plus. 2023;5:100040. 39802172 10.1016/j.jmccpl.2023.100040 PMC11708335

[61] Caliandro R, Husetić A, Ligtermoet ML, Hanemaaijer-van der Veer J, van Duijvenboden K, Zentilin L, Clerkx M, Giacca M, Oostra RJ, van den Hoff MJB, Christoffels VM, Gladka MM. AAV6-based ZEB2 delivery promotes cardiomyocyte dedifferentiation in adult human myocardium. Cardiovasc Res. 2025;121(16):2462–4. 41071925 10.1093/cvr/cvaf190 PMC12713634

[62] Kiselev E, Agyapong W, Jurgens B, Mohr E, Chatterjee S, Hunkler HJ, et al. Transcriptional and functional effects of mavacamten in multiple porcine and human models with hypertrophic cardiomyopathy. Br J Pharmacol. 2026;183(5):1122–39. 41215595 10.1111/bph.70247

[63] Watson SA, Terracciano CM, Perbellini F. Myocardial Slices: an Intermediate Complexity Platform for Translational Cardiovascular Research. Cardiovasc Drugs Ther. 2019;33(2):239–44. 30671746 10.1007/s10557-019-06853-5 PMC6509068

[64] Halbach M, Pillekamp F, Brockmeier K, Hescheler J, Muller-Ehmsen J, Reppel M. Ventricular slices of adult mouse hearts–a new multicellular in vitro model for electrophysiological studies. Cell Physiol Biochem. 2006;18(1–3):1–8. 16914885 10.1159/000095132

[65] Kang C, Qiao Y, Li G, Baechle K, Camelliti P, Rentschler S, et al. Human Organotypic Cultured Cardiac Slices: New Platform For High Throughput Preclinical Human Trials. Sci Rep. 2016;6:28798. 27356882 10.1038/srep28798 PMC4928074

[66] Khan MS, Shahid I, Bennis A, Rakisheva A, Metra M, Butler J. Global epidemiology of heart failure. Nat Rev Cardiol. 2024;21(10):717–34. 38926611 10.1038/s41569-024-01046-6

[67] Conrad N, Judge A, Tran J, Mohseni H, Hedgecott D, Crespillo AP, et al. Temporal trends and patterns in heart failure incidence: a population-based study of 4 million individuals. Lancet. 2018;391(10120):572–80. 29174292 10.1016/S0140-6736(17)32520-5 PMC5814791

[68] Pilcher LE, Hancock E, Neeli A, Sckolnick M, Caporizzo MA, Palmer BM, et al. Loss of Snord116 protects cardiomyocyte kinetics during ischemic stress. J Mol Cell Cardiol Plus. 2025;11:100291. 40124788 10.1016/j.jmccpl.2025.100291 PMC11928973

[69] Abbas N, Bentele M, Waleczek FJG, Fuchs M, Just A, Pfanne A, et al. Ex vivo modelling of cardiac injury identifies ferroptosis-related pathways as a potential therapeutic avenue for translational medicine. J Mol Cell Cardiol. 2024;196:125–40. 39341589 10.1016/j.yjmcc.2024.09.012 PMC7617241

[70] Cardinale D, Iacopo F, Cipolla CM. Cardiotoxicity of Anthracyclines. Front Cardiovasc Med. 2020;7:26. 32258060 10.3389/fcvm.2020.00026 PMC7093379

[71] Cardinale D, Colombo A, Lamantia G, Colombo N, Civelli M, De Giacomi G, et al. Anthracycline-induced cardiomyopathy: clinical relevance and response to pharmacologic therapy. J Am Coll Cardiol. 2010;55(3):213–20. 20117401 10.1016/j.jacc.2009.03.095

[72] Schmidt K, Fuchs M, Weber N, Werlein C, Schmitto JD, Ius F, et al. Single-nucleus RNA sequencing identifies cell-type-specific effects of sodium-glucose co-transporter 2 inhibitors in human myocardial slices. Eur Heart J. 2024;45(35):3292–5. 39082743 10.1093/eurheartj/ehae472 PMC11400937

[73] Witman N, Zhou C, Grote Beverborg N, Sahara M, Chien KR. Cardiac progenitors and paracrine mediators in cardiogenesis and heart regeneration. Semin Cell Dev Biol. 2020;100:29–51. 31862220 10.1016/j.semcdb.2019.10.011

[74] Van Linthout S, Stellos K, Giacca M, Bertero E, Cannata A, Carrier L, et al. State of the art and perspectives of gene therapy in heart failure. A scientific statement of the Heart Failure Association of the ESC, the ESC Council on Cardiovascular Genomics and the ESC Working Group on Myocardial & Pericardial Diseases. Eur J Heart Fail. 2025;27(1):5–25. 39576264 10.1002/ejhf.3516 PMC11798634

[75] Wang J, Jonker T, Cervera-Barea A, Dong Z, Visser RN, Birza EE, et al. AAV6 vectors provide superior gene transfer compared to AAV9 vectors following intramyocardial administration. Mol Ther Methods Clin Dev. 2025;33(3):101532. 40777723 10.1016/j.omtm.2025.101532 PMC12329528

[76] Gladka MM, Kohela A, Molenaar B, Versteeg D, Kooijman L, Monshouwer-Kloots J, et al. Cardiomyocytes stimulate angiogenesis after ischemic injury in a ZEB2-dependent manner. Nat Commun. 2021;12(1):84. 33398012 10.1038/s41467-020-20361-3 PMC7782784

